# Lateral Cephalometric Radiography: Principles, Common Positioning Errors, and AI-Driven Quality Control

**DOI:** 10.3390/diagnostics16040543

**Published:** 2026-02-12

**Authors:** Rossana Izzetti, Maria Pisano, Chiara Cinquini, Lorenzo Cinci, Antonio Barone, Cosimo Nardi

**Affiliations:** 1 Unit of Dentistry and Oral Surgery, Department of Surgical, Medical, and Molecular Pathology and Critical Care Medicine, University of Pisa, 56126 Pisa, Italy; 2 Department of Experimental and Clinical Biomedical Sciences, University of Florence, 50121 Florence, Italy

**Keywords:** cephalometry, orthodontics, artificial intelligence, cone-beam computed tomography

## Abstract

This narrative review provides a contemporary synthesis of lateral cephalometric radiography (LCR), addressing both its foundational principles and the impact of technological integration, with a focus on enhancing diagnostic reliability. A structured literature search (PubMed, up to September 2025) was conducted around five domains: LCR’s diagnostic role, acquisition methods, positioning errors, comparisons with cone-beam computed tomography (CBCT), and Artificial Intelligence (AI)-driven quality control. Precise patient positioning—maintaining symmetry and a horizontal Frankfort plane—is paramount, as common errors (tilting, rotation, nodding) introduce quantifiable inaccuracies in key measurements. While digital innovation, particularly deep learning models for automated landmark detection and error flagging, improves the consistency of workflow, current AI tools require validation and human oversight to manage limitations in generalizability. When contextualized against three-dimensional imaging, LCR maintains a favorable balance of diagnostic utility and lower radiation dose, supporting its selective, indication-based use in contemporary practice. Ultimately, this review suggests that adherence to a meticulous acquisition technique remains the cornerstone of reliable LCR analysis, even as AI and digital tools evolve to augment the clinician’s role.

## 1. Introduction

The lateral cephalometric radiograph (LCR) has been an integral part of orthodontic practice since its introduction in 1931, establishing a standardized two-dimensional (2D) representation of the craniofacial complex for diagnosis and treatment planning [1,2]. For decades, it has provided a critical tool for evaluating skeletal, dental, and soft tissue relationships. Despite its foundational role, the clinical utility and technical execution of LCR are subjects of ongoing debate and evolution [3,4]. While indispensable for quantitative assessment in many complex cases, inherent limitations of conventional 2D radiography—including projective geometric errors, sensitivity to patient positioning, landmark identification variability, and radiation exposure concerns, especially in pediatric populations—have prompted scrutiny of its routine necessity [5]. Concurrently, the technological landscape has transitioned from film-based systems to digital acquisition, and more recently, toward the integration of artificial intelligence (AI) for automated analysis and quality control [6]. However, this rapid technological shift, while promising enhanced efficiency and consistency, introduces new challenges regarding algorithmic validation, generalizability, and integration into established clinical workflows.

At present, a significant gap exists in the literature that synthesizes these parallel narratives: the importance of foundational acquisition principles and the potential of digital innovation. While technological advances are often documented, there is a need for a cohesive analysis of how innovations like AI-driven automation interact with—and can potentially mitigate—traditional sources of error to redefine the modern diagnostic pathway.

This narrative review, therefore, addresses this gap with a dual focus. First, it provides a detailed examination of the core principles of LCR image acquisition and a systematic analysis of common patient positioning errors, quantifying their impact on measurement accuracy. Second, it critically evaluates the latest technological advancements to analyze how these tools are creating a novel diagnostic workflow. By bridging fundamental knowledge with forward-looking innovation, this review aims to serve as a comprehensive and practical guide for clinicians and radiologists navigating the evolving standards of contemporary orthodontic practice.

## 2. Methods

The research was conducted by two groups of reviewers (Group 1: RI/MP/CC; Group 2: LC/AB/CN). Both groups attended an online meeting to ensure alignment during the article selection process. The literature search was structured around five domains, namely the role of LCR in orthodontics, acquisition methods, sources of error during LCR execution, comparisons with cone-beam computed tomography (CBCT), and AI-driven methods aimed at ensuring optimal image acquisition. The two groups of reviewers independently conducted a double Medline search via PubMed, including literature published up to September 2025. All relevant articles identified were discussed between the groups prior to inclusion in the review. No predefined or restrictive keyword strategy was applied.

## 3. The Role of LCR in Orthodontic Treatment Planning

LCR provides a reproducible, quantitative, and standardized assessment of craniofacial structures, which is useful for monitoring the direction and magnitude of facial growth, assessing dentoalveolar development, and accurately quantifying the skeletal and soft-tissue changes induced by orthodontic treatment over time [7,8]. The technique’s primary benefit lies in its serial comparability, enabled by the standardization of patient positioning using a cephalostat. This repeatability is critical for longitudinal evaluations, allowing clinicians to monitor progress [8]. Beyond skeletal and dental analyses, a correctly executed LCR enables the evaluation of the nasopharyngeal airway, cervical spine morphology, and hyoid bone position—structures intrinsically linked to craniocervical posture and respiratory function, which can influence craniofacial development [9]. Achieving this level of diagnostic accuracy is dependent upon a technically optimal image acquisition. The standardized geometry of the cephalostat, with fixed distances between the X-ray tube, the patient’s head (centered on the sella turcica), and detector, is designed to minimize geometric magnification and distortion, ensuring that linear and angular measurements remain consistent across serial radiographs [10].

However, the decision to obtain an LCR necessitates a careful risk–benefit analysis, balancing its diagnostic potential against radiation exposure, particularly in pediatric patients [11]. The evidence regarding its indispensability for orthodontic treatment planning is still debated [12].

The contradictory findings in the literature can be attributed to several factors, including heterogeneity in study samples (e.g., mix of simple and complex malocclusions), variability in clinician experience, and differences in methodological design [13].

Ultimately, LCR’s impact is not universal but is dictated by its role in specific clinical decision-making steps [14,15]. Therefore, the current evidence supports a selective, indication-based approach where LCR provides decisive quantitative data precisely where clinical judgment reaches its limits.

Studies indicate that the influence of LCR is case-dependent. For example, Devereux et al. [2] reported that LCR caused a fundamental change to the treatment plan, primarily in patients with skeletal Class II malocclusions, while Nijkamp et al. [13] found no such effect in cases of Class II/Division 1 malocclusion. Similarly, some studies found LCR altered planning in a limited number of cases [16,17], whereas others, like those by Dinesh et al. [3] and Durao et al. [12], reported minimal impact on final decisions in the majority of evaluated cases. This suggests that LCR’s value is most pronounced in differentiating between the dental and skeletal etiology of a malocclusion, quantifying the severity of a sagittal or vertical skeletal discrepancy, assessing the soft tissue profile and its relationship to underlying skeletal structures, and determining the need for and timing of growth modification or surgical intervention [17].

## 4. Technology and Acquisition Methods

**Figure 1:**
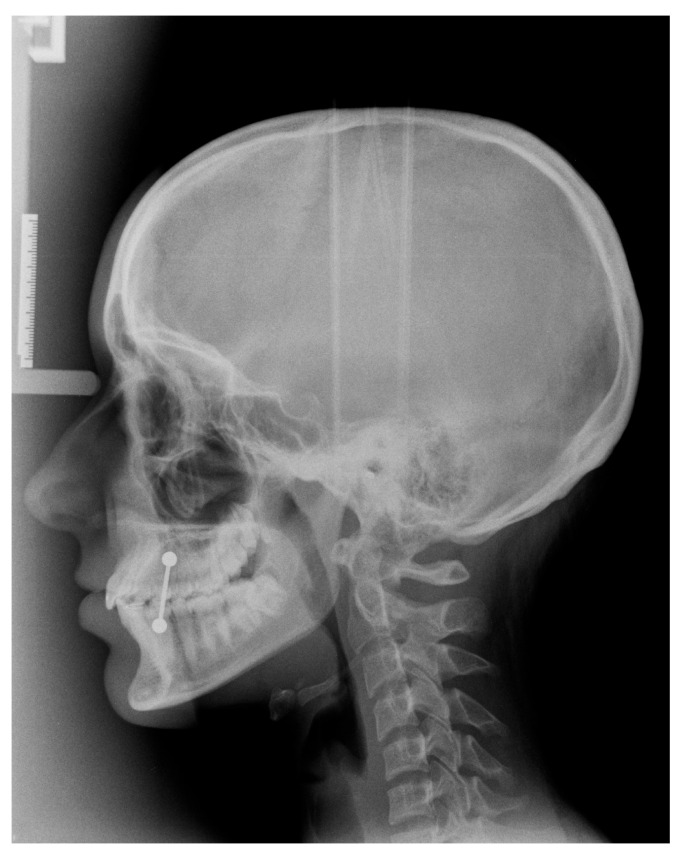
Representative lateral cephalometric radiograph acquired using an indirect digital device (photostimulable phosphor plate system), demonstrating the full craniofacial field of view, including vertex and occiput. Acquisition parameters: 90 kVp, 15 mA, 0.8 s.

The image acquisition methodology in LCR is defined by the type of detector, with significant implications for image quality, artifact potential, and clinical workflow. In analog (film-screen) or computed radiography (photostimulable phosphor plate) systems, a single, brief exposure captures the entire field of view instantaneously [18]. This “single shot” method is fast, minimizing the risk of motion blur. Single-shot systems typically capture the entire skull, including the vertex and occiput (Figure 1).

Early direct digital radiography (DR) systems often utilize a moving sensor bar that scans the craniofacial structures with a narrow radiation beam over several seconds (typically 3–6 s). Direct DR systems utilize a moving sensor bar that scans the craniofacial structures with a narrow radiation beam over several seconds [19]. This prolonged scanning time increases the risk of motion artifacts, which can degrade landmark clarity. Therefore, patient instruction and cooperation are more critical when using DR systems to ensure a still posture is maintained throughout the extended exposure. Importantly, the finite travel range of the sensor bar often truncates the posterior or superior skull regions, a limitation clinicians must acknowledge during interpretation [20]. While modern cephalometric analyses rarely require these peripheral structures, this limitation of DR should be considered, particularly in cases where cranial base assessment is needed [21,22]. DR detectors, however, increasingly employ large-format, single-shot flat-panel detectors that acquire the image instantaneously, similarly to computed radiography, thereby eliminating scan-time artifacts. Modern orthopantomography units with integrated cephalostats can automatically adjust the field of view based on patient anatomy, partially mitigating this issue [22,23].

The exposure technique in LCR is optimized for both diagnostic yield and patient safety. The standard use of a high-kilovoltage (“hard beam”) technique (100–110 kVp) provides the moderate contrast necessary to visualize both bony landmarks and the soft tissue profile simultaneously, which is essential for comprehensive analysis [24,25,26]. In pediatric patients, dose-optimization protocols may employ lower kVp settings while maintaining diagnostic quality. This technique also contributes to dose reduction, as fewer photons deposit their full energy in patient tissues [25,26]. While this can result in images that appear overexposed, digital post-processing allows for easy correction to achieve diagnostic quality—a significant advantage over analog methods where overexposure would render an image non-diagnostic [23]. Adherence to the ALARA (As Low As Reasonably Achievable) principle is paramount, especially for the pediatric and adolescent demographic, which constitutes the majority of orthodontic patients. This involves justifying each examination, employing optimized digital systems, and utilizing the exposure parameters that deliver the minimum dose required for an adequate diagnostic image [27].

LCR can also be acquired using hybrid imaging devices that combine panoramic, cephalometric, and cone-beam computed tomography (CBCT) capabilities in a single unit [28]. While offering workflow efficiency, the CBCT components in such hybrid devices often feature small fields of view suited for dentoalveolar imaging rather than full craniofacial capture. Dedicated large-field CBCT scanners, whether standalone or, less commonly, integrated into hybrids, can reconstruct synthetic 2D cephalograms from a 3D volumetric dataset. Although this provides a projection-error-free image, the associated radiation dose is typically higher than that of a standard 2D LCR, necessitating strict clinical justification [29].

## 5. Correct Execution

**Figure 2:**
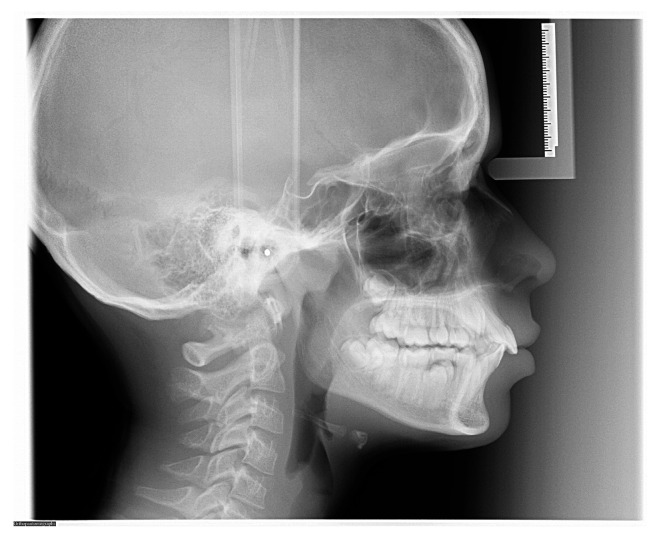
Properly acquired lateral cephalometric radiograph using a direct digital radiography (DR) system with a scanning sensor bar. Patient positioning: standing; Frankfurt plane horizontal; midsagittal plane parallel to the detector; teeth in maximum intercuspation with lips closed. Central ray centered on the external auditory meatus. There is perfect superimposition of the bilateral radiopaque ear rod markers, confirming absence of tilt or rotation. Acquisition parameters: 100 kVp, 20 mA, scanning time ~4 s. The field of view is truncated superiorly and posteriorly, as is typical for DR systems.

In LCR, the X-ray tube and detector are positioned at a standard distance of 1.524 m (5 feet), while the patient-to-detector distance is 15 cm. The consistent distance between the X-ray tube and the center of the patient’s head and between the center of the patient’s head and the detector limits intrinsic geometric distortions in two-dimensional imaging, particularly the magnification effect. This makes it possible to obtain repeatable measurements over time to compare examinations performed on the same patient at different stages of treatment [11]. The midpoint between the ear rods is considered the central point of the skull and corresponds to the sella turcica. From this central reference point, the block supporting the ear rods can be rotated to perform postero-anterior cephalometric projection. Specifically, the positioning for the frontal cephalogram is identical to that for the lateral plane, except the patient’s head is rotated 90 degrees towards the detector. LCRs allow for the visualization of the soft tissues of the face as there is a wedge filter at the X-rays source that absorbs part of the radiation, thereby making soft tissues perceptible in the radiograph (Figure 2).

The imperative for correct execution extends to patient safety. The diagnostic necessity of the examination must always be weighed against the principle of As Low As Reasonably Achievable (ALARA), particularly given that a significant proportion of orthodontic patients are children and adolescents [12]. Modern digital acquisition systems contribute to this balance by optimizing exposure parameters, often employing a higher-kilovoltage (“hard beam”) technique. This approach reduces patient dose while providing the moderate contrast necessary for delineating both bone landmarks and the soft tissue profile—a dual requirement for comprehensive cephalometric analysis [8,24]. Therefore, this meticulous technique not only safeguards the diagnostic reliability and reproducibility of the LCR but also aligns with the ethical obligation to minimize radiation exposure.

## 6. Errors in Patient Positioning During LCR

**Figure 3:**
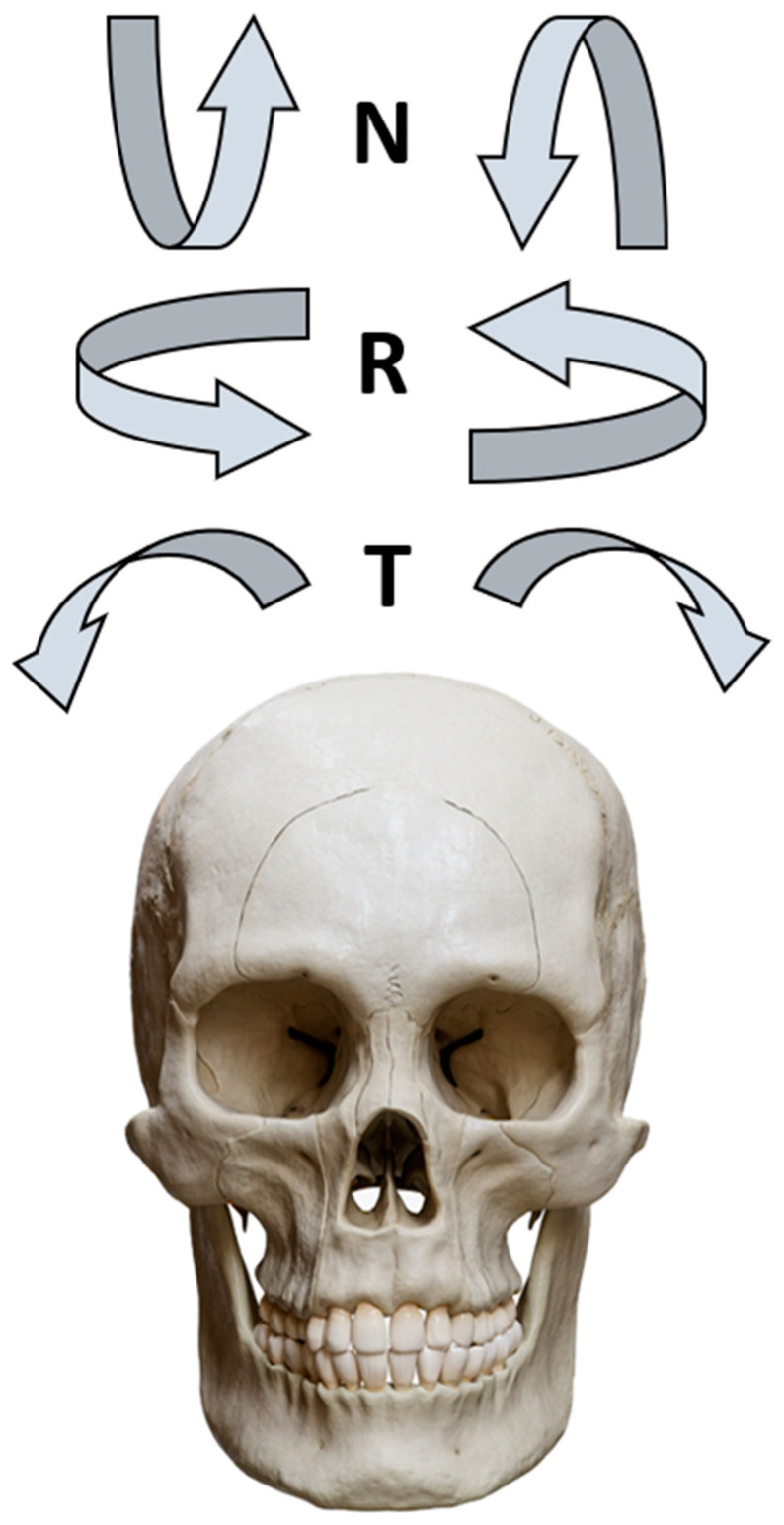
Schematic illustration of the three primary types of patient head movement during cephalometric acquisition: nodding (N, pitch around the transverse axis), rolling (R, rotation around the vertical axis), and tilting (T, movement along the coronal plane).

Positioning errors do not merely reduce image clarity; they systematically distort the projected relationships between craniofacial landmarks, leading to quantifiable inaccuracies in the angular and linear measurements that form the basis of cephalometric diagnosis and treatment planning. Specifically, tilting refers to lateral movement along the coronal plane; rolling refers to rotation around the vertical axis; and nodding refers to pitch movement around the transverse (inter-aural) axis. Such errors can be categorized as follows and are schematized in Figure 3:

- Lateral head movement (Tilting): Movement along the coronal plane, leading to asymmetrical images and difficulty assessing bilateral structures.
- Head rotation (Rolling): Rotation around the vertical axis, distorting midline structures and bilateral superimposition.
- Chin elevation or depression (Nodding): Alters the vertical relationships of facial structures, affecting the mandibular plane angle and facial height assessment.
- Misalignment of the Frankfurt plane: Results in incorrect orientation of the craniofacial structures relative to the true horizontal.
- Failure to achieve maximum intercuspation: The mandible must be in its most superior occlusal position where teeth are fully interlocked; failure to do so invalidates the assessment of arch relationships.

**Figure 4:**
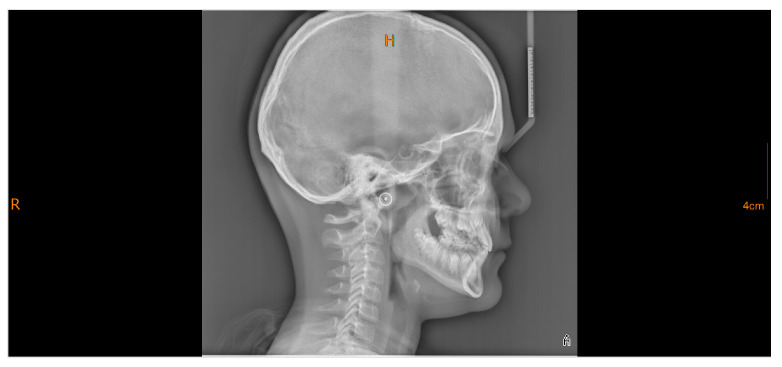
Lateral cephalometric radiograph acquired using a hybrid imaging device (combined panoramic/cephalometric/CBCT unit). Note the distinct radiopaque target marker (outer circle with central point) overlying each external acoustic meatus, with the acquisition parameters of 90 kVp, 15 mA, and 0.8 s indicating precise alignment of the ear rods and correct patient positioning without tilt or rotation.

Radiopaque markers on the ear rods and frontonasal support are visible on the radiograph to verify correct positioning. Properly positioned ear rods appear as a single overlapping point or a distinct target image, indicating the absence of tilting or rolling, while the frontonasal support prevents nodding (Figure 4) [30,31]. Motion during exposure may manifest as blurred dental structures.

### 6.1. Horizontal Plane

**Figure 5:**
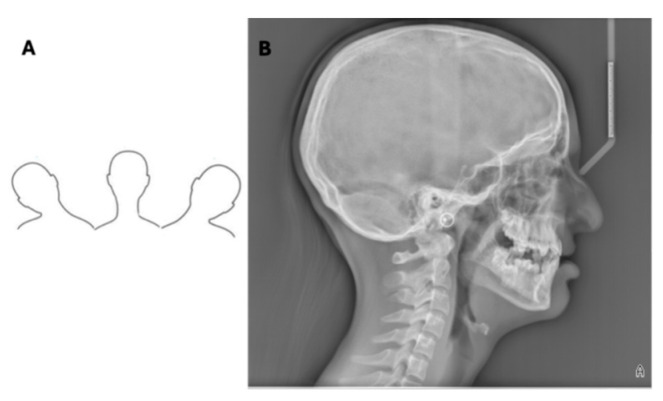
Tilting. (**A**) Picture. (**B**) LCR performed with hybrid device. Head tilt results in asymmetric projection of craniofacial structures of the right and left sides. These structures are not overlapping on the radiograph but misaligned in the cranio-caudal direction. Such misalignment is evident at the molar teeth and mandibular bone profiles. In addition, note the open positioning of the lips. Acquisition parameters: 90 kVp, 15 mA, 0.8 s.

**Figure 6:**
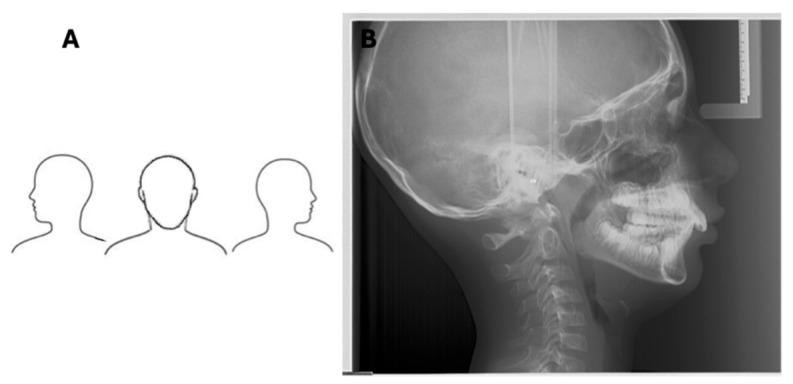
Rolling. (**A**) Picture. (**B**) LCR. Acquired with direct digital device. Head roll results in the asymmetric projection of craniofacial structures of the right and left sides. These structures do not appear overlapping on the radiograph but are misaligned in the antero-posterior direction. Such misalignment is evident at the molar teeth and mandibular bone profiles. Acquisition parameters: 100 kVp, 20 mA, scanning time ~4 s.

An asymmetric head position on the horizontal plane typically results from incorrect placement of the ear rods. When the radiopaque markers are perfectly superimposed, a symmetric position is achieved [32]. Asymmetry due to patient positioning, rather than true anatomy, is evidenced by a lack of superimposition of the posterior mandibular rami, orbits, and external acoustic meati. Tilting displaces these structures in the cranio-caudal direction, while rolling causes misalignment in the antero-posterior direction (Figure 5 and Figure 6).

### 6.2. Sagittal Plane

**Figure 7:**
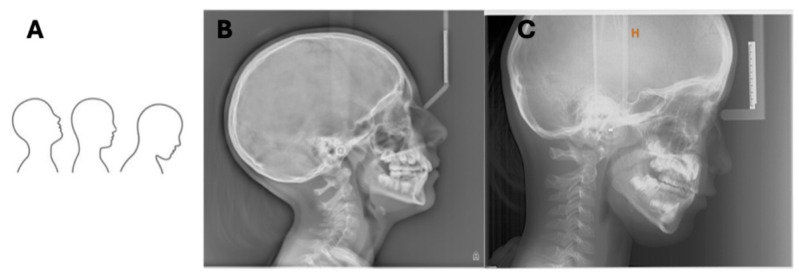
Nodding. (**A**) Picture. (**B**) LCR carried out via hybrid device. (**C**) LCR carried out via direct digital device. Nodding up and nodding down are favored by the misalignment of the spine according to the axis with the ear rods, resulting in an emphasis on cervical lordosis or straightening of the cervical spine. Acquisition parameters for hybrid device are as follows: 90 kVp, 15 mA, 0.8 s. Acquisition parameters for direct digital device are as follows: 90 kVp, 15 mA, 0.8 s.

Errors in the sagittal plane involve upward or downward head movement (nodding). The Frankfurt plane must be horizontal and parallel to the floor to project symmetric bilateral structures at the same height. Misalignment leads to nodding, which is often associated with compensatory spinal posture, resulting in an emphasis on cervical lordosis or straightening of the cervical spine (Figure 7) [33]. The frontonasal support is crucial for stabilizing the antero-posterior position of the head.

### 6.3. Occlusal Plane

**Figure 8:**
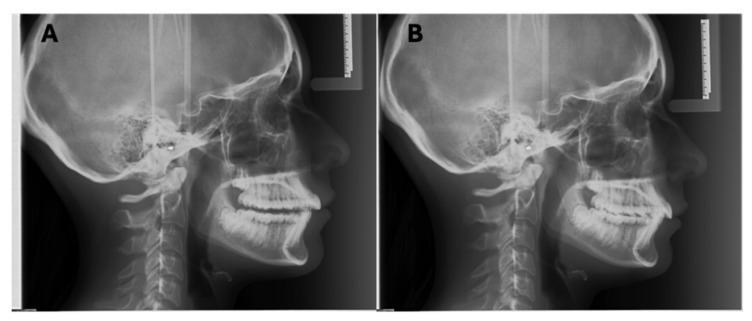
LCR carried out via direct digital device and necessarily repeated for failure to maximum intercuspation (**A**). The second attempt shows correct intercuspation (**B**). Acquisition parameters: 90 kVp, 15 mA, 0.8 s.

**Figure 9:**
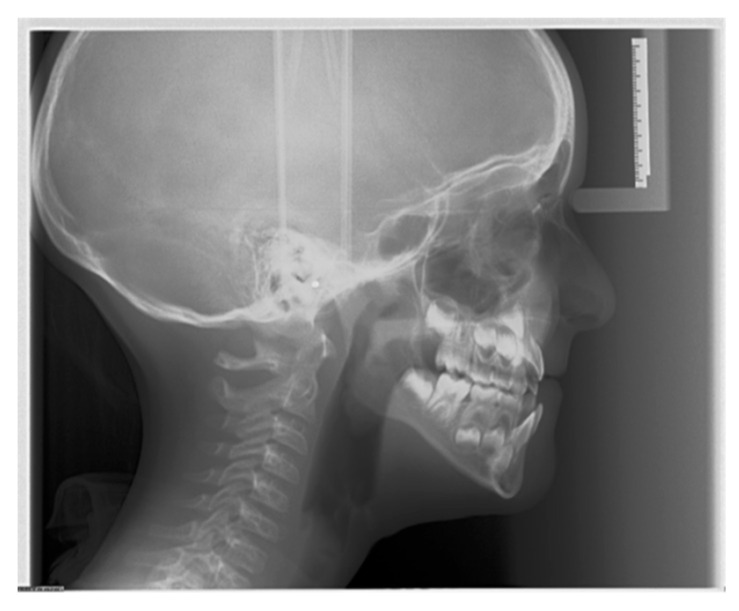
LCR carried out via direct digital device with lips tightened. Such positioning error is only detected with state-of-the-art devices that enable a good representation of facial soft tissues. Acquisition parameters: 90 kVp, 15 mA, 0.8 s.

Patients must maintain maximum intercuspation during exposure (Figure 8). Failure to achieve this necessitates a repeat examination to accurately assess the relationship between the upper and lower dental arches [32]. To facilitate this, patients should perform opening and closing movements, swallow, and then maintain a natural lip seal—neither separated nor pursed (Figure 9)—during the exposure. It is acknowledged that patients with malocclusions or craniofacial anomalies may not have a stable intercuspation; in such cases, retaking the LCR would only be harmful due to unjustified re-exposure.

To achieve a correct occlusion with dental crowns in maximum intercuspation, it is beneficial for the patient to perform some mouth-opening and -closing movements beforehand, then swallow and finally maintain occlusion during the radiation exposure, keeping the lips naturally closed. The lips should be in a competent position, neither separated nor pursed.

### 6.4. Cervical Spine

**Figure 10:**
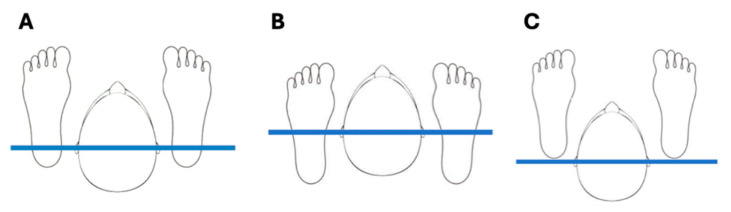
Ear rods–malleoli axis. (**A**) Correct placement of the patient’s ankles in relation to the ear rods. Malleoli should correspond to the vertical projection to the ground of the external acoustic meati. (**B**) Incorrect positioning of the feet forward, with malleoli placed anteriorly to the external acoustic meati. (**C**) Incorrect positioning of the feet backward, with malleoli placed posteriorly to the external acoustic meati. Failure to comply with the ear rods–malleoli axis leads to an incorrect spinal column positioning, which in turn facilitates—but does not oblige—nodding the head upwards or downwards in case ankles are located anteriorly or posteriorly to the ear rods, respectively.

The alignment of the cervical spine and associated structures is critical for a standardized, reproducible head posture [34]. The “ear rods–malleoli axis” is a key mechanical principle: positioning the patient’s ankles (malleoli) directly under the vertical projection of the ear rods to the floor aligns the body’s weight-bearing axis, promoting a neutral, unstrained cervical posture (Figure 10A). Incorrect foot placement either anterior or posterior to this axis (Figure 10B,C) facilitates compensatory nodding of the head forward or backward, respectively, to re-establish balance, thereby altering the cervical spine’s radiographic representation.

### 6.5. Impact of Positioning Errors on Cephalometric Analysis

Head positioning errors can significantly impact the accuracy of cephalometric measurements, causing the incorrect identification of key landmarks like sella turcica, nasion, and gonion, which are critical anatomical references for assessing skeletal relationships [35].

These effects, such as a lateral head rotation (rolling) of 5°, can result in discrepancies of over 2° in the ANB angle and 2 mm in the Wits appraisal, while a 10° pitch (nodding) can alter the mandibular plane angle (SN-GoGn) by more than 3° and anterior facial height by up to 4 mm [22,30,32,33,35]. These changes can be clinically significant, potentially shifting diagnostic classifications (e.g., from skeletal Class I to Class II) or altering surgical treatment plans. A synthesis of quantified impacts from key studies is provided in Table 1.

Positioning errors become particularly problematic when comparing serial radiographs for growth assessment or treatment monitoring. Emerging AI-based tools are being developed to address positioning errors. Deep learning models, particularly convolutional neural networks (CNNs), are trained to detect specific error patterns [36]. Algorithms can specialize in identifying asymmetry in bilateral structures or identifying nodding by assessing the angle of the Frankfort plane or cervical spine alignment relative to a learned horizontal standard [18]. Other systems offer real-time feedback via preview monitors, allowing for repositioning before exposure, while post-processing software can algorithmically correct minor rotational (rolling) misalignments by digitally re-orienting the image to a standardized reference plane [37,38]. These AI-driven methods aim to reduce operator dependency and improve consistency, although their clinical integration requires validation across diverse patient cohorts and imaging devices. A checklist to minimize positioning errors is proposed in Table 2, and additional recommendation for the clinician is provided in Table A1 and Table A2.

## 7. Comparative Analysis of LCR, CBCT, and AI-Driven Innovations

In recent years, LCR has evolved from analog film-based systems into sophisticated digital platforms, offering enhanced resolution, lower radiation exposure, and excellent diagnostic accuracy [39]. The implementation of digital sensors and high-definition imaging software for 2D imaging has improved landmark visibility and minimized geometric distortion, facilitating more accurate evaluations [39,40]. However, its flat geometry may still be inadequate for studying anatomical volume and its evolution during growth. Consequently, even in orthodontics, CBCT has been significantly increasing in the last few years since this technique is capable of achieving precise reconstructions of bone structures in all three dimensions of space [41]. The advent of CBCT has introduced a new dimension to orthodontic imaging, offering an accurate visualization of complex anatomical relationships [42]. While CBCT eliminates projection errors and magnification artifacts, providing 86.4% accuracy in landmark identification within 1 mm for a majority of clearly defined craniofacial landmarks [43], it exposes patients to higher radiation doses than conventional radiographs. Reported ranges vary widely from approximately 50–600 µSv for CBCT versus 5–20 µSv for LCR, depending on device settings, field of view, and patient age [44,45]. It is important to note that these numeric comparisons are influenced by significant heterogeneity across studies, including differences in CBCT machine types, acquisition protocols, dosimetry methods, and landmark definitions. Furthermore, the cited accuracy percentage for CBCT landmark identification is derived from studies with specific sample characteristics and imaging conditions; generalizability may be limited by variations in operator experience, image quality, and anatomical complexity. Therefore, the role of CBCT in orthodontic diagnosis and treatment planning is still being evaluated due to the increased cost and interpretation time and the lack of standardized 3D cephalometric norms. Given that orthodontic patients are often children and adolescents, who are more sensitive to the effects of ionizing radiation, it is essential to weigh the potential benefits of CBCT against the risks of radiation exposure.

The most transformative innovation, however, is the integration of AI, which has revolutionized cephalometric analysis.

Contemporary AI tools are powered by sophisticated deep learning architectures. Modern Convolutional Neural Networks (CNNs) can achieve sub-2 mm mean radial error for landmark identification on standardized datasets, matching the intra-observer consistency of trained clinicians [44,45]. However, AI performance is contingent on the quality, size, and diversity of training datasets. Models trained on specific populations or imaging devices may suffer from reduced generalizability (dataset bias) and can struggle with atypical anatomy, severe malocclusions, or poor-quality images—scenarios where clinical expertise remains irreplaceable [46,47]. Therefore, AI currently functions best as an assistive tool, with human oversight being essential for verification, especially in complex cases.

Concurrently, cloud-based storage and digital workflow platforms have modernized data management, enabling the secure, real-time sharing of cephalometric records across multidisciplinary teams. This is particularly beneficial in complex treatments requiring coordination between various specialists and in the growing field of tele-orthodontics [48]. Evidence on the accuracy of digital setups and the influence of 3D predicted outcomes on patient expectations underscores the clinical implications of adopting digital cephalometric workflows and the need to validate predictive models against real outcomes [49]. While CBCT-based volumetric assessments can reveal anatomical relationships that are not visible on 2D radiographs [50], advanced algorithms—often generative adversarial networks (GANs) or other deep learning models trained on paired 2D–3D data—can now extrapolate pseudo-3D models from standard LCR, offering enhanced anatomical insight without increasing radiation burden [51]. These reconstructions are inferential approximations, not true measurements, and their accuracy is constrained by the inherent loss of 3D information in a 2D projection, limiting their current clinical utility [52,53].

The integration of AI into clinical practice also introduces ethical and regulatory considerations, including algorithmic transparency, data privacy, and the need for rigorous clinical validation to meet medical device standards [54].

Critically, these reconstructions are inferential approximations rather than true measurements; their accuracy is fundamentally constrained by the loss of 3D information in a 2D projection, making them unreliable for assessing transverse dimensions or asymmetries and limiting their validation primarily to research settings [52].

Collectively, these innovations are reshaping clinical practice and advancing orthodontic education and research, as digital platforms enable interactive learning, large-scale data analysis, and the development of predictive diagnostic tools. Nevertheless, the field must address key challenges—including algorithmic bias, data privacy in cloud systems, and the rigorous clinical validation of AI and pseudo-3D tools—to ensure these technologies are both equitable and robust. As digital technology continues to progress, LCR remains an essential and increasingly powerful instrument in modern orthodontic diagnostics, defined by its precision, efficiency, and adaptability [55].

## 8. Conclusions

Lateral cephalometric radiography remains a useful diagnostic tool in orthodontics when applied selectively and with appropriate clinical justification. The accuracy and reproducibility of cephalometric analysis are strongly dependent on correct image acquisition, as patient positioning errors can substantially affect landmark identification and measurement reliability, particularly in longitudinal evaluations.

Advances in digital imaging and artificial intelligence have improved workflow efficiency and consistency of analysis; however, they do not replace the need for standardized technique and careful patient positioning. AI-based tools may support landmark detection and quality control, but their clinical application still requires validation and professional oversight. Future progress hinges on establishing robust, multi-center validation pathways for AI tools and developing standardized cephalometric norms derived from 3D datasets to better inform 2D diagnostic criteria.

Compared with three-dimensional imaging, LCR continues to offer a favorable balance between diagnostic information and radiation exposure. While cone-beam computed tomography provides additional spatial detail, its routine use remains limited by the higher radiation doses and restricted indications. This narrative review is inherently limited by its methodological design, which, while comprehensive, does not constitute a systematic synthesis and may reflect the selective inclusion of evidence. However, LCR appears to retain a defined role in contemporary orthodontic practice when used according to indication-based criteria and established radiological principles.

## Figures and Tables

**Table 1:** Quantified impact of common positioning errors on cephalometric measurements.

Positioning Error Type	Quantified Error Induced	Key Cephalometric Parameters Affected	Primary Clinical Implication
Head Rotation (Rolling)	5° rotation from midsagittal plane	ANB angle (>2° change), Wits appraisal (>2 mm change), mandibular midline shift	Misdiagnosis of sagittal skeletal relationship (Class I/II/III); altered symmetry assessment [30,32]
Head Tilt (Tilting)	5° tilt in coronal plane	Mandibular plane asymmetry, cant of occlusal plane, vertical heights of bilateral structures	False positive diagnosis of facial asymmetry; incorrect vertical analysis [32,35]
Head Pitch (Nodding Up/Down)	10° pitch from Frankfort horizontal	SN-GoGn/FMA (>3° change), anterior facial height (N-Me, up to 4 mm change), SNB angle	Altered vertical skeletal diagnosis (hyper-/hypodivergent); affects surgical planning and growth assessment [33,35]
Failure in Maximum Intercuspation	Open bite posture (2–3 mm)	Overjet/overbite measurement error, incisor inclination, lower anterior face height	Inaccurate assessment of dentoalveolar relationships and occlusal plane [22,32]

**Table 2:** Practical checklist for LCR patient positioning. A well-positioned LCR ensures reliable measurements and valid serial comparisons. When in doubt, reposition—do not rely on post-processing correction.

Step	Goal/Action	Common Pitfall to Avoid	Verification Step/Action If Failed
1. Head Stabilization	Secure ear rods in external auditory meati; adjust frontonasal support.	Loose fit allows tilting/rolling; avoid patient discomfort that causes movement.	Check for a snug, non-painful fit. If markers are not aligned on preview, re-seat ear rods.
2. Frankfort Plane	Ensure plane (porion-orbitale) is horizontal and parallel to the floor.	Chin up/down (nodding) skews vertical measurements and mandibular plane angle.	Visually confirm horizontal alignment using the cephalostat guide or spirit level. Reposition if plane is oblique.
3. Midsagittal Alignment	Align patient’s midline parallel to detector; left side faces detector.	Head rotation (rolling) distorts bilateral symmetry and midline landmarks.	Check for symmetrical projection of bilateral structures (e.g., orbital rims). Rotate head until midline is parallel.
4. Occlusion and Lips	Instruct: “Bite in maximum intercuspation, lips lightly closed.”	Open bite posture or strained lips alter soft-tissue profile and occlusal assessment.	On preview, check for contact between upper and lower incisors. Ask patient to swallow and re-close if open.
5. Whole-Body Posture	Position ankles (malleoli) under ear rods; ensure relaxed neck and shoulders.	Leaning forward/backward promotes compensatory head nodding and cervical spine distortion.	Visually align malleoli with the vertical drop line from the ear rods. Adjust stance if misaligned.
6. Final Verification	Check for superimposed ear-rod markers on preview; confirm patient stillness.	Proceed if markers overlap as a single point/target; retake if blur or asymmetry is suspected.	Do not expose unless markers are perfectly superimposed. If blur is present, repeat instruction and expose.

## Data Availability

Data sharing is not applicable (no new data were generated).

## Appendix A

**Table A1:** Evidence-based recommendations for clinical practice.

Aspect	Key Recommendations	Rationale/Evidence Base
Imaging Modality Selection: LCR vs. CBCT	**Favor Standard LCR for:** Initial diagnosis and treatment planning for most malocclusions. Longitudinal growth assessment and treatment monitoring. Cases requiring profile (soft-tissue) analysis. **Consider CBCT for:** Severe craniofacial asymmetry. Impacted/collapsed canines (localized FOV). Planning for orthognathic surgery or TAD placement. Cases where 3D airway or TMJ analysis is critical. **Always apply the ALARA principle and use the lowest dose necessary for diagnosis**	LCR provides sufficient diagnostic data for most orthodontic plans with a significantly lower radiation dose (5–20 µSv) vs. CBCT (50–600 µSv). CBCT is justified when 3D spatial information is essential for the treatment decision and cannot be obtained from 2D records.
Patient Positioning Protocol (Condensed Checklist)	**Stabilize:** Firmly seat ear rods in external auditory meati; adjust frontonasal support. **Align Planes:** Ensure Frankfurt plane (porion-orbitale) is horizontal; align midsagittal plane parallel to detector. **Secure Occlusion:** Instruct patient to bite in maximum intercuspation, with lips lightly closed. Have patient swallow just prior to exposure. **Stabilize Posture:** Position ankles (malleoli) under the vertical line of the ear rods; ensure relaxed neck/shoulders. **Verify and Expose:** Check for perfect superimposition of ear-rod markers on preview image. Confirm patient stillness and expose.	Systematic positioning errors (tilt, rotation, nodding) introduce quantifiable measurement errors (>2° change in ANB, >3° in MP angle) that compromise diagnosis and serial comparison. A standardized protocol minimizes these errors.
Integration of AI Tools	**Use AI for:** Automated landmark detection to improve intra-/inter-observer consistency. Automated quality control to flag potential positioning errors. **Always maintain human oversight** to verify AI outputs, especially in complex/atypical cases, and interpret clinical significance.	AI models can match expert accuracy for landmark identification but may fail on atypical anatomy or poor-quality images. They are assistive tools that require clinical validation and expert judgment.

**Table A2:** Checklist to ensure precise, reproducible, and diagnostically reliable LCR acquisitions. *When in doubt, reposition—do not rely on post-processing correction*.

Step	Goal/Action	Key Verification
Head Stabilization	Firmly seat ear rods in external auditory meati. Adjust frontonasal support for gentle contact.	Fit is snug but not painful. Ear rod markers are centered in preview.
Frankfort Plane Alignment	Align porion-orbitale plane horizontally, parallel to the floor.	Visual/spirit level check. Plane must not be oblique; reposition if tilted.
Midsagittal Alignment	Position patient’s midline parallel to detector; left side faces detector.	Bilateral structures (orbital rims) appear symmetrical in preview.
Occlusion and Lips	Instruct: “Bite in maximum intercuspation, lips lightly closed.” Have patient swallow just before exposure.	Incisors are in contact on preview. No lip strain or separation.
Whole-Body Posture	Place ankles (malleoli) under the vertical drop-line of the ear rods. Shoulders and neck relaxed.	Feet are not forward or backward; patient stands comfortably upright.
Final Verification and Exposure	Confirm perfect superimposition of ear-rod markers (single point/target). Ensure patient is still.	DO NOT EXPOSE if markers are doubled or blurred. Reposition and retry.
